# Prediction by a multiparametric magnetic resonance imaging-based radiomics signature model of disease-free survival in patients with rectal cancer treated by surgery

**DOI:** 10.3389/fonc.2024.1255438

**Published:** 2024-02-22

**Authors:** Jiwei Mao, Wanli Ye, Weili Ma, Jianjiang Liu, Wangyan Zhong, Hang Yuan, Ting Li, Le Guan, Dongping Wu

**Affiliations:** ^1^ Department of Radiation Oncology, Shaoxing People’s Hospital, Shaoxing, China; ^2^ Department of Radiology, Shaoxing People’s Hospital, Shaoxing, China

**Keywords:** rectal cancer, radiomics, prognosis, disease-free survival, MRI

## Abstract

**Objective:**

The aim of this study was to assess the ability of a multiparametric magnetic resonance imaging (MRI)-based radiomics signature model to predict disease-free survival (DFS) in patients with rectal cancer treated by surgery.

**Materials and methods:**

We evaluated data of 194 patients with rectal cancer who had undergone radical surgery between April 2016 and September 2021. The mean age of all patients was 62.6 ± 9.7 years (range: 37–86 years). The study endpoint was DFS and 1132 radiomic features were extracted from preoperative MRIs, including contrast-enhanced T1- and T2-weighted imaging and apparent diffusion coefficient values. The study patients were randomly allocated to training (n=97) and validation cohorts (n=97) in a ratio of 5:5. A multivariable Cox regression model was used to generate a radiomics signature (rad score). The associations of rad score with DFS were evaluated using Kaplan–Meier analysis. Three models, namely a radiomics nomogram, radiomics signature, and clinical model, were compared using the Akaike information criterion.

**Result:**

The rad score, which was composed of four MRI features, stratified rectal cancer patients into low- and high-risk groups and was associated with DFS in both the training (*p* = 0.0026) and validation sets (*p* = 0.036). Moreover, a radiomics nomogram model that combined rad score and independent clinical risk factors performed better (Harrell concordance index [C-index] =0.77) than a purely radiomics signature (C-index=0.73) or clinical model (C-index=0.70).

**Conclusion:**

An MRI radiomics model that incorporates a radiomics signature and clinicopathological factors more accurately predicts DFS than does a clinical model in patients with rectal cancer.

## Introduction

Being one of the major causes of cancer-related death worldwide, rectal cancer is a global health problem (1, 2). About 70% of rectal cancer patients are successfully treated surgically. However, the prognosis is poor, distant metastases or local recurrence being detected in up to 30% of patients, often within a few years of surgery (3, 4). Identifying tumor characteristics that are associated with a high-risk of adverse outcomes is therefore extremely important in enabling devising risk-adapted personalized treatment strategies for rectal cancer patients.

Nowadays, the TNM classification is routinely used in clinical practice for preoperative risk stratification and treatment allocation (5, 6). However, this classification ignores tumoral spatial heterogeneity, which indicates hemorrhage, necrosis, and cell density and thus provides important guidance for decisions concerning administration of radio- and/or chemo-therapy. Radiomics involves extracting a large set of quantitative features from a series of radiological images. In particular, radiomics based on multiparametric MRI (mpMRI) has emerged as a reproducible, non-invasive means of characterizing intratumor features and assessing risk and treatment response (7).

To the best of our knowledge, only one study on using mpMRI, diffusion kurtosis imaging in particular, to predict the response to therapy in patients with rectal cancer has been published (8). Because this is rarely performed in clinical practice, there are no established standard scan parameters; thus, its generalizability and usefulness need to be further verified. Diffusion weighted imaging (DWI), a functional MRI technique, has been widely used in clinical practice.

The apparent diffusion coefficient (ADC), which is derived from DWI sequencing, reflects heterogeneity at the microscopic level, such as cell density of tumors (9). The aim of our study was to establish a model for predicting the disease-free survival (DFS) of rectal cancer treated by surgery by combining contrast-enhanced (CE)-T1WI, T2WI, ADC radiomics features, and clinical factors.

## Methods

### Patients

This study was approved by our institution’s ethics review board, who waived the need for patient approval and informed consent because this was a retrospective study.

This study cohort comprised 194 patients (121 men and 73 women; mean age: 62.6 ± 9.7 years; age range: 37–86 years) with histologically confirmed rectal cancer who had undergone resection in our institution between April 2016 and September 2021. The inclusion criteria were as follows: (a) preoperative conventional MRI and DWI sequences performed within 3 weeks before resection; (b) pathologically confirmed rectal cancer; and (c) follow-up at our hospital. Exclusion criteria were: (a) administration of preoperative chemoradiotherapy; (b) another cancer in addition to rectal cancer; and (c) failure to attend for follow up.

All patients were randomly allocated to training (n=97) and validation groups (n=97) in a ratio of 5:5.

### Follow-up

We set the DFS, defined as the time from CT examination until either the date of disease progression, including distant metastasis, local tumor recurrence, or death from any cause, or until the last date known to be free of relapse (censored) as the main endpoint. All disease progression was diagnosed on the basis of findings on imaging such as abdominal CT and MRI, clinical examination, or biopsy. Our institution’s follow-up protocol is every 3–6 months during the first 2 years after surgery, every 6–9 months for the following 2–3 years, and annually thereafter. The median follow-up period of the whole cohort was 32 months (range, 4–99 months).

### Image acquisition

Rectal MRI imaging was obtained on a 3T system (Verio, Siemens, Germany) equipped with a 12-channel body coil. The MRI scanning sequence included high-resolution axial T2-weighted, DWI, and enhanced T1-weighted sequences. Detailed information concerning the acquisition parameters is provided in **Supplementary Materials**.

### Tumor segmentation and feature extraction

**Figure 1:**
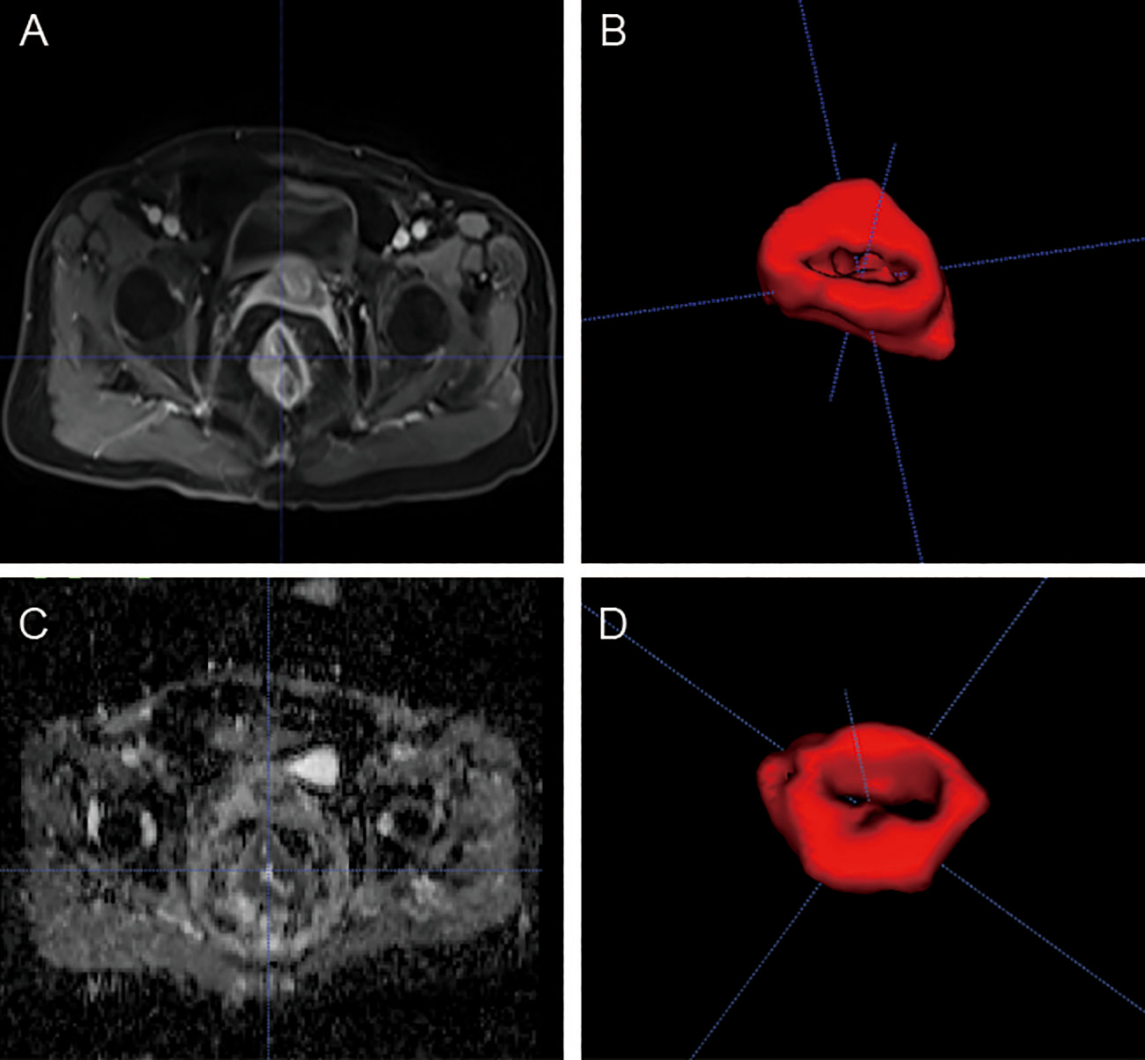
MR images of a 53-yearold man with rectal cancer. Examples of tumors with MRI **(A, C)** and 3D segmentation **(B, D)**.

Manual segmentation of each tumor was performed using open-source ITK-SNAP software (www.itksnap.org) on data obtained from axial T2WI, ADC, and CE-T1WI slices. Region of interest (ROI) segmentation was performed by two experienced radiologists who examined the whole tumor and avoided necrotic tissue and bleeding (**Figure 1**).

Radiomics feature extraction was preprocessed by a pyradiomics package (http://www.PyRadiomics.readthedocs.io/en/latest/) that comprises four groups of features. In total, 1132 radiomic features, including ROI shape, intensity, texture, and wavelet features, were then extracted from multiple image sequences from each patient. To obtain a standardized normal distribution of the MRI image intensities, multiple images were normalized by z-score after manual ROI segmentation of the tumor.

### Radiomic feature selection and radiomics signature building

We used a four-step process for feature selection and to identify robust DFS-associated radiomic features. Intraclass correlation coefficients of >0.75 were considered to denote high inter-/intra-observer stability and kept for subsequent analyses. Univariate Cox regression analyses were then conducted to identify statistically significant DFS-related radiomic features (p ≤ 0.05). Next, Spearman’s correlation analysis with r≥0.90 was used to eliminate redundancy. Finally, multivariate Cox analysis was performed to develop independent predictors of DFS. Radiomics signatures (defined as rad scores) were computed through a linear combination of each selected feature with non-zero coefficients.

### Statistical analysis

All statistical analyses were analyzed with R software, version 3.6.3 (http://www.R-project.org). Continuous and categorical variables were compared between the training and validation sets by using an independent samples *t*-test, Mann–Whitney U-test, or χ^2^ test as appropriate. DFS probabilities were assessed by Kaplan–Meier analysis and the differences between high- and low-risk groups were compared with the log-rank test. The optimal cutoff value according to the rad score and X-tile was used to divide patients into low- and high-risk groups. The Harrell concordance index (C-index) was calculated to quantify the model’s ability to discriminate. Decision curve analysis was used to evaluate the clinical usefulness of various models. A two-sided *p*<0.05 was regarded as denoting statistical significance.

## Results

### Clinical characteristics and DFS

Selected clinico-radiological characteristics of the 194 study patients are shown in **Table 1**.

**Table 1:** Clinical characteristics of patients with rectal cancer in the training data set and validation data set.

Characteristic	Training set(n=97)	Validation set(n=97)	*P*
Age (years)(mean±SD)	63.4±8.8	62.0±10.5	0.468
Sex			
male	61	60	0.882
female	36	37	
CEA (ng/ml)			
≥5.5	38	43	0.467
<5.5	59	54	
CA199 (ng/ml)			
≥30	76	75	0.863
<30	21	22	
Ki67	40(30,50)	70(45,70)	0.126
Tumor differentiation			0.198
Well	10	8	
Mediate	67	58	
Poor	20	31	
Adjuvant chemotherapy			
Yes	12	16	0.414
No	85	81	
tumor size(mm)	64.3±20.6	43.4±16.1	0.525
MRF			
Positivity	59	47	0.351
Negativity	38	40	
EVI			
Positivity	13	10	0.505
Negativity	84	87	
pT			
T1	7	7	0.925
T2	21	23	
T3	47	47	
T4	22	18	
pN			
N0	63	70	0.362
N1	20	19	
N2	14	8	
Follow-up time (mo)	36.3±22.4	38.1±23.3	0.591

MRF, mesorectal fascia; EVI, extramural venous invasion.

**Figure 2:**
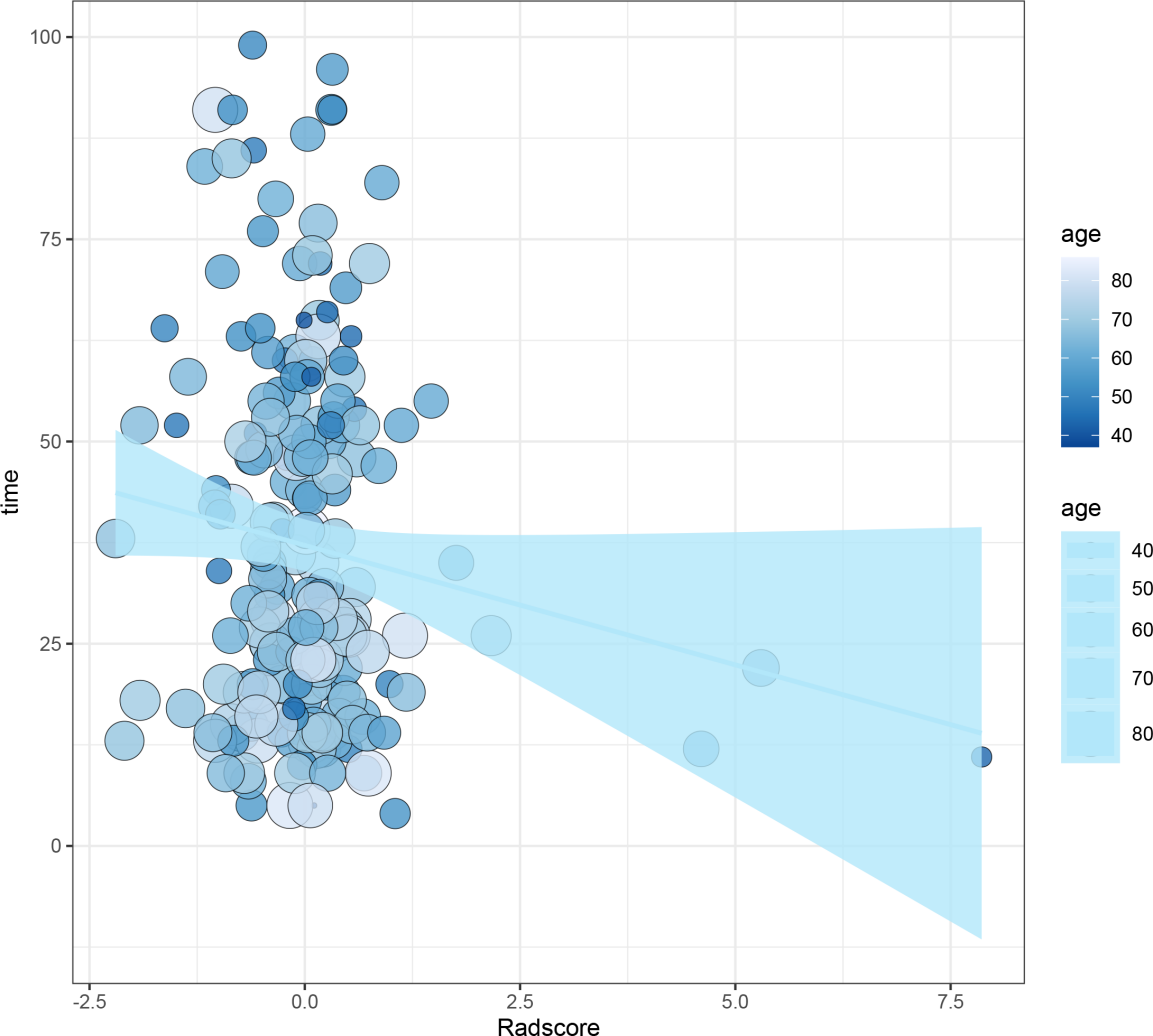
The relationships between survival time, age, and rad-score.

The median follow-up period of the whole cohort was 32 months (range, 4–99 months). The relationships between survival time, age, and rad-score are shown in **Figure 2**.

### Radiomics score construction and validation of radiomics signature

Four potential predictors including one, one, and two features were selected from the T2WI, ADC and CE-T1WI to build a radiomics signature based on a formula for calculating radiomics score (**Supplementary Materials**).

**Figure 3:**
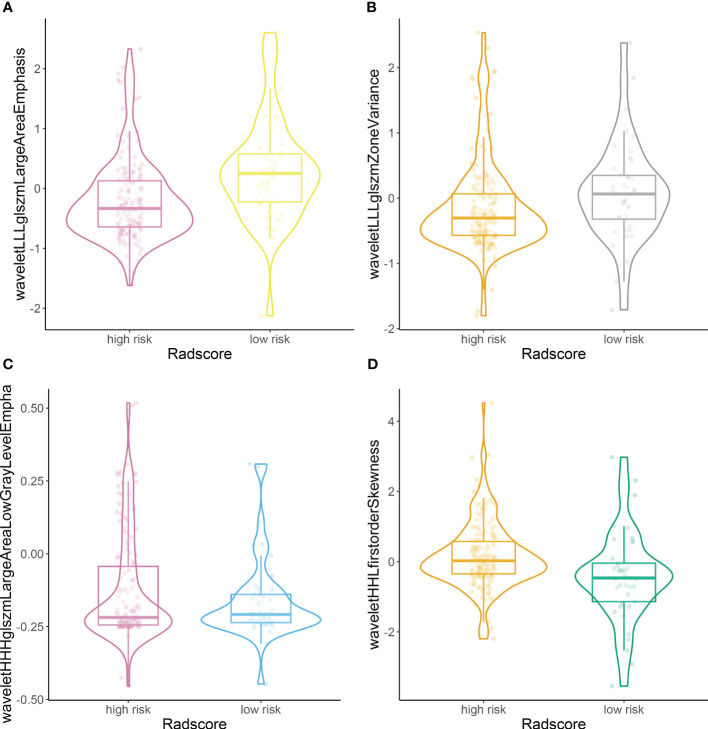
Plots **(A–D)** illustrate the distributions of the high- and low-risk rad scores of the included four features.

According to the rad score optimum cut-off point generated by X-tile plot, we further classified patients into high- (rad score ≥ −0.7) and low-risk groups (rad-score < −0.7), and performed Kaplan–Meier analysis in the training and validation sets to determine the ability of the rad score to predict prognosis. The distributions of the high- and low-risk rad scores of the included four features are shown in **Figure 3**.

**Figure 4:**
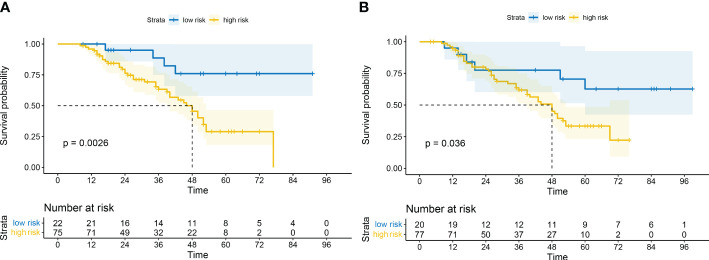
Kaplan–Meier survival analysis based on high risk and low risk rad scores in training set **(A)** and validation set **(B)**.

Lower rad scores were associated with better DFS in both the training (*p* = 0.0026) and validation sets (*p* = 0.036) (**Figure 4**).

### Assessment of the radiomics nomogram model on DFS prediction

**Figure 5:**
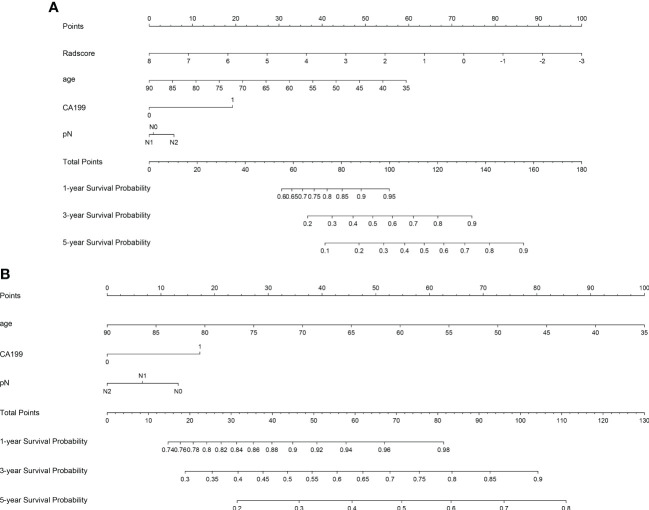
Plots illustrate radiomics nomogram **(A)** and clinical model nomogram **(B)** for the prediction of predicting DFS.

Three models (radiomics nomogram, clinical, radiomics signature) were assessed in the training and validation sets. The ability of each model to discriminate was then evaluated by the C-index in the validation set. Only three clinical features, namely age, CA199, and pN, were used in the clinical model. The abilities of the radiomics nomogram and clinical models to predict DFS are shown in **Figure 5**. The clinical and radiomics signature models alone had similar discriminatory capability (C-index: 0.70 vs. 0.73). However, a radiomics nomogram model incorporating the radiomics signature and clinical model was better able to discriminate DFS in the validation cohort (C-index, 0.77) than was either the radiomic signature or clinical model alone (**Table 2**).

**Table 2:** Performance of models.

Model	Training set		Validation set		AIC
	C-index	95%CI	C-index	95%CI	
Radiomics nomogram	0.76	0.71-0.79	0.77	0.71-0.82	781.5
Radiomics signature	0.72	0.69-0.75	0.73	0.70-0.78	793.4
Clinical model	0.71	0.68-0.75	0.70	0.69-0.79	788.6

AIC, Akaike information criterion.

**Figure 6:**
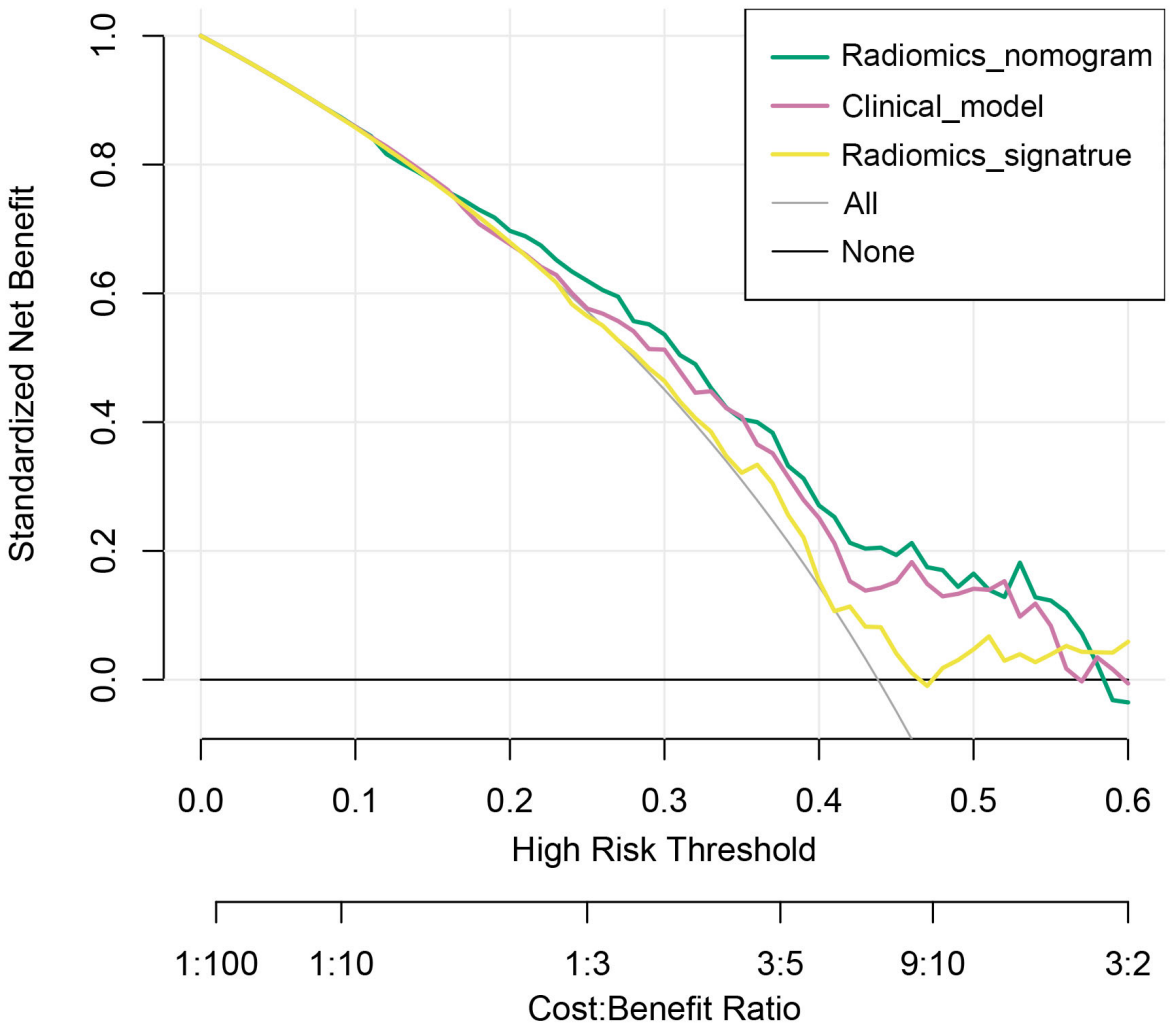
Decision curve analysis of the radiomics nomogram model and clinical model.

Decision curve analysis also revealed that the radiomics nomogram model achieved a higher net benefit than did the clinical model or radiomics signature in predicting DFS (**Figure 6**).

The cumulative 3-year DFS rates were 42.7% versus 63.6%, respectively, (*p* < 0.05), for high- versus low-risk patients in the training set, and 48.1% versus 60.0%, respectively, (*p* < 0.05) in the validation set. Subsequently, the cumulative 5-year DFS rates were 10.7% versus 36.4%, respectively, (*p* < 0.05), for high- versus low-risk patients in the training set and 13.0% versus 45.0%, respectively, (*p* < 0.05) in the validation set.

## Discussion

Radiomics features, which are noninvasively acquired high-dimension features from radiological images, are closely associated with treatment response (10), prognosis (11), and molecular phenotypes (12). Previous studies have found that radiomics can predict individual responses to neoadjuvant therapy for rectal cancer (13–15). Several studies have shown that radiomics models have promising prognostic value in patients with rectal cancer (8, 16). However, because diffusion kurtosis imaging is rarely used in clinical practice, there is a lack of standard scan parameters. This means that the generalizability and clinical relevance of using radiomics models based on features identified by this modality to predict responses to therapy require further verification. Furthermore, the above-cited studies did not examine all stages of rectal cancer treated by surgery.

Giving this background, we constructed a multi-feature-based radiomics signature extracted from mpMRI to evaluate the prognostic value of radiomics in patients with rectal cancer. Our clinical and radiomics signature models had similar discriminatory capability (C-index: 0.70 vs. 0.73). However, a combination of the radiomics nomogram model incorporating the radiomics signature and our clinical model improved the discriminating power for DFS in the validation cohort, as evidenced by a higher C-index (0.77), lower Akaike information criterion (781.5), and improvement in reclassification. This is in line with previous research (13, 17, 18) and demonstrates that the radiomics signature has an incremental value in risk stratification for predicting DFS.

Additionally, rad scores can stratify patients into high- and low-risk groups. Lower rad scores (< −0.7) in patients with rectal cancer were generally associated with better DFS, suggesting that some high-risk patients should receive risk-adapted personalized treatment strategies such as neoadjuvant chemoradiotherapy or adjuvant chemoradiotherapy. Moreover, rad scores were significantly associated with DFS (*p*<0.05), showing that they can be a useful tool for individualized estimation of survival of patients with rectal cancer.

Intriguingly, the four radiomics features selected for the integrated radiomics model were all wavelet-based, which is consistent with recent studies (19–21). This shows that almost all high-dimensional features are wavelet-based. Wavelet transformation enables quantification of high-dimensional tumor information, which is difficult to explain intuitively (21, 22). In one study (7), the researchers constructed two types of radiomics signatures: with and without wavelet features. However, there was no evidence that the model with wavelet features improved the accuracy of prediction; this may be attributable to redundant radiomics features (23). Most high-dimensional features are not perceptible visually; however, they have been used successfully in radiomics-based prediction of survival (24, 25), recurrence (26), and gene expression (27).

The present study had several limitations. First, the sample size was relatively small. Second, because this was a retrospective, single-center study, our findings need to be further validated by drawing prospectively on a large-scale multicenter database. Finally, stratification of other risk factors derived from staging and pathologic type may provide more accurate estimation of risk of survival and recurrence.

In conclusion, mpMRI radiomics improves prediction of prognosis in patients with rectal cancer. Our comprehensive model including clinical features provides additional prognostic information beyond a clinical model alone. Further prospective studies and clinical validation are required.

## Data availability statement

The original contributions presented in the study are included in the article/**Supplementary Material**. Further inquiries can be directed to the corresponding author.

## Ethics statement

The studies involving humans were approved by Academic Committee of Shaoxing People’s Hospital. The studies were conducted in accordance with the local legislation and institutional requirements. The participants provided their written informed consent to participate in this study. Written informed consent was obtained from the individual(s) for the publication of any potentially identifiable images or data included in this article.

## Author contributions

JM: Writing – original draft. WY: Writing – original draft. WM: Methodology, Writing – original draft. JL: Software, Writing – original draft. WZ: Software, Writing – original draft. HY: Visualization, Writing – original draft. TL: Visualization, Writing – original draft. LG: Writing – original draft. DW: Writing – original draft.
