# Supplementary material for: Prediction by a multiparametric magnetic resonance imaging-based radiomics signature model of disease-free survival in patients with rectal cancer treated by surgery
Source: Front Oncol. 2024 Feb 22;14:1255438. doi: 10.3389/fonc.2024.1255438 (PMC10917947; doi:10.3389/fonc.2024.1255438)
Supplement: Supplementary file 1 [file DataSheet_1.docx]

All imaging data were obtained using a 3.0-T MRI scanner (Verio, Siemens, Germany) with a 12-channel phased-array body coil. Patients were required to fast for at least 8 h to empty the gastrointestinal tract and inject antispasmodic medication (Anisodamine, Minsheng, Hangzhou, China) before the MRI examination to reduce gastrointestinal artifacts. During the MRI scan, the patient was placed in the supine position, and the positioning line was located on the xiphoid process. All patients underwent a routine plain scan (T1WI, T2WI with fat suppression, T2WI, ADC, DWI) before the CE-T1WI scan and a multi-angle cross-sectional T1WI in the axial plane scan (repetition time/echo time, 3.48 ms/1.3 ms; layer thickness, 3 mm; field of view, 260 mm × 260 mm; matrix, 202 × 288). CE-T1WI, the contrast agent was meglumine gadolinate, the total amount was 0.1 mmol/kg. After 40 seconds of injection, image acquisition was performed, the parameters were TR 3.56 ms, TE 1.39 ms, FOV 34.9 cm × 34.9 cm, NSA=1, layer thickness 4.5 mm, no layer spacing.
